# Mg-Incorporated Nickel Oxide Hole Injection Layer for Stable and Efficient Quantum Dot Light-Emitting Diodes

**DOI:** 10.1021/acs.jpclett.5c02298

**Published:** 2025-08-28

**Authors:** Meng-Wei Wang, Ting Ding, Yin-Man Song, Hang Liu, Jing Jiang, Pei-Li Gao, Kar Wei Ng, Shuang-Peng Wang

**Affiliations:** 59193 Institute of Applied Physics and Materials Engineering, University of Macau, Taipa, Macao SAR 999078, China

## Abstract

High-quality hole injection layers (HILs) are essential for efficient and stable quantum dot light-emitting diodes (QLEDs). While NiO_*x*_ is a stable alternative to the poly­(3,4-ethylenedioxythiophene):poly­(styrenesulfonate) (PEDOT:PSS) HIL, its low hole injection limits its practical application. This work enhances NiO_*x*_ hole injection efficiency by combining Mg alloying to deepen work function (5.49 eV vs 5.20 eV) with O_3_ treatment to boost conductivity while suppressing traps. Using sol–gel synthesized Mg-alloyed NiO_*x*_ nanoparticles followed by O_3_ treatment via atomic layer deposition, the resulting QLEDs achieve peak efficiencies of 17.85 cd A^–1^ and 11.23 lm W^–1^, representing 54% and 171% improvements over NiO_*x*_-based QLEDs (11.56 cd A^–1^, 4.15 lm W^–1^). Operational stability significantly improves, with a *T*_50_ lifetime of 272 h (*L*_0_ = 1000 cd m^–2^), over 2.2-fold that of NiO_*x*_-based QLEDs (84 h). This methodology provides a viable pathway to develop a NiO_*x*_ HIL for advancing stable and high-efficiency QLEDs.

## Graphical abstract

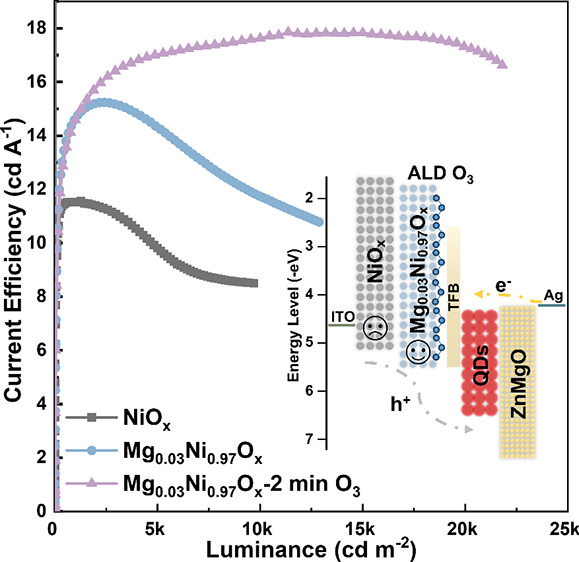

Quantum dot light-emitting diodes (QLEDs), distinguished by exceptional color purity, broad gamut coverage, and high electroluminescence (EL) efficiency, have emerged as a leading candidate for next-generation display technologies and advanced lighting systems.^1–3^ Notably, the external quantum efficiency (EQE) of red, green, and blue QLEDs has approached the theoretical limit of light output coupling, reaching 36.5%,^4^ 28.7%,^5^ and 21.9%,^5^ respectively. State-of-the-art QLEDs utilize an organic–inorganic hybrid architecture,^6^ where the conjugated polymer poly­(3,4-ethylenedioxythiophene):poly­(styrenesulfonate) (PEDOT:PSS) serves as the predominant hole injection layer (HIL) owing to its high work function, high conductivity, and superior optical transparency.^7,8^ However, the acidity and hygroscopicity of PSS chains severely compromise the stability of the device under storage and operating conditions.^9,10^

Intrinsically robust transition metal oxides, including NiO_*x*_, MoO_3_, and V_2_O_5_, are widely employed as HIL replacements of PEDOT:PSS.^11–14^ Among them, nonstoichiometric NiO_*x*_ is notable for its intrinsic p-type semiconductor characteristics,^15,16^ arising from nickel vacancy-induced acceptor levels that promote hole formation via electron transitions from the valence band maximum (VBM). Furthermore, the VBM exhibited in NiO_*x*_ facilitates a stepped energy level alignment that enhances energy level matching, while its wide bandgap (*E*_g_ > 3.50 eV)^17^ confers a robust blocking ability for leakage electrons. However, the wide-bandgap-induced low conductivity (10^–7^ S cm^–1^),^18^ coupled with the still substantial energy offset (0.2–0.6 eV)^19^ between the Fermi level of NiO_*x*_ and the highest occupied molecular orbital (HOMO) energy level of the conventional hole transport layer (HTL), jointly hinder the hole injection efficiency of NiO_*x*_-based QLEDs. Recent years have witnessed numerous strategies (Ji et al.,^20^ Wan et al.,^21^ Cao et al.,^22^ Wang et al.^23^) aimed at designing the device architecture and modifying the hole injection in NiO_*x*_-based QLEDs. Sun et al.^24^ achieved excellent performance by inserting an ultrathin insulating layer of LiF between QDs and NiO_*x*_ to balance the charge injection in QLEDs and avoid charging of QDs. In addition, Sargent et al.^25^ revealed that the molecular orientation of self-assembled monolayers (SAMs) regulates the energy level of NiO_*x*_. SAMs containing a trifluoromethyl group deepened the work function of NiO_*x*_ to improve the energy level alignment. Despite these advances, the incorporation of functional layers still undesirably introduces imperfect interfaces, and the mechanism underlying the high hole efficiency of NiO_*x*_ HIL remains unclear, necessitating further investigation.

In this work, we demonstrate an interface engineering strategy combining Mg-alloying of NiO_*x*_ nanoparticles (Mg:NiO_*x*_ NPs) to deepen the work function with precisely controlled O_3_ treatment to enhance the conductivity. This synergistic approach significantly boosts the hole injection capability of the NiO_*x*_ HIL, enabling efficient and stable QLEDs. Specifically, Mg:NiO_*x*_ NPs synthesized via a sol–gel method exhibit a work function of 5.49 eV, significantly deeper than that of pristine NiO_*x*_ NPs (5.20 eV). Furthermore, subsequent O_3_ treatment of the Mg_0.03_Ni_0.97_O_*x*_ film precisely controlled via atomic layer deposition reduces trap states and further enhances hole conductivity. As a result, highly efficient Mg_0.03_Ni_0.97_O_*x*_-2 min O_3_ QLEDs are achieved with an EL peak at 620 nm, a peak current efficiency of 17.85 cd A^–1^, and a peak power efficiency of 11.23 lm W^–1^, which are significantly improved by 54% and 171% compared with control NiO_*x*_-based QLEDs (11.56 cd A^–1^, 4.15 lm W^–1^), respectively. Meanwhile, Mg_0.03_Ni_0.97_O_*x*_-2 min O_3_ QLEDs exhibit a 272 h *T*_50_ lifetime (*L*_0_ = 1000 cd m^–2^), which is over 2.2-fold that of the NiO_*x*_-based QLEDs (84 h). This study offers a promising strategy for designing a NiO_*x*_ HIL to develop more stable and efficient lighting and display technologies.

NiO_*x*_ and Mg:NiO_*x*_ NPs were prepared via a sol–gel method. The thermogravimetric analysis (TGA) confirms complete decomposition of Ni­(CH_3_COO)_2_·4H_2_O and Mg­(CH_3_COO)_2_·4H_2_O precursors at 400 °C within 1 h (Figure S1). The X-ray diffraction (XRD) was performed to interrogate the crystallinity of NiO_*x*_ and Mg:NiO_*x*_ films (Figure 1a). The three peaks at 37.18°, 43.30°, and 62.82° can be attributed to the (111), (200), and (220) crystal planes of NiO_*x*_ and Mg:NiO_*x*_ NPs, indicating a face-centered cubic (FCC) structure, which is highly consistent with the standard pattern of NiO_*x*_ (JCPDS-NiO Card no.78-0643).^26^ Furthermore, the absence of MgO peaks indicates that Mg is incorporated into the NiO_*x*_ lattice. Detailed comparison of the (200) diffraction peaks (Figure 1b) reveals a low angle shift from 43.30° to 43.15° as the Mg composition increases from 0 to 0.04, attributed to the larger ionic radius of Mg^2+^ (72 pm) compared to Ni^2+^ (69 pm).^27^

**Figure 1:**
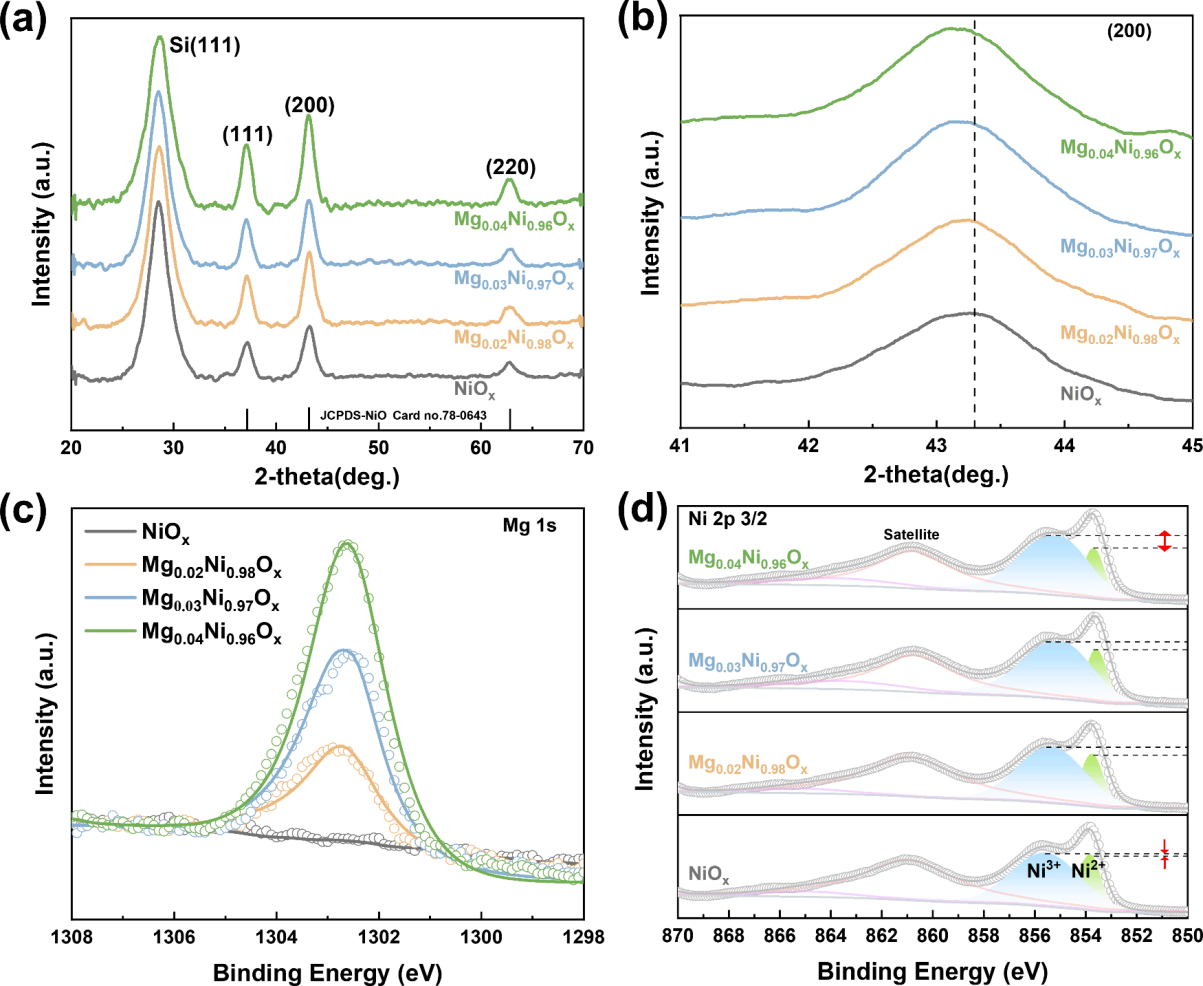
Properties of Mg_*y*_Ni_1–*y*_O_*x*_ NPs. (a) XRD spectra and (b) detailed comparison of (200) diffraction peaks. (c) XPS spectra of Mg 1s and (d) Ni 2p_3/2_ peaks of Mg_*y*_Ni_1–*y*_O_*x*_ films.

The surface chemical states and compositions of Mg_*y*_Ni_1–*y*_O_*x*_ NPs were studied by X-ray photoelectron spectroscopy (XPS) analysis (Figures 1c and S2). The Mg 1s photoelectron peaks at 1302.7 eV exhibit progressively enhanced intensity with an increase in Mg composition, confirming the successful incorporation of Mg into the NiO_*x*_ film. This finding aligns with the surface chemical compositions reported previously for Mg_*y*_Ni_1–*y*_O_*x*_ films.^28^ From the Ni 2p_3/2_ spectra (Figure 1d), the Mg_*y*_Ni_1–*y*_O_*x*_ films can be deconvoluted and fitted to the peaks corresponding to the Ni^2+^ (853.8 eV) and Ni^3+^ (855.4 eV) oxidation states. The incorporation of Mg leads to an increase in the Ni^3+^/Ni^2+^ ratio from 2.57 to 3.95 with a higher Mg composition (Table 1). This Ni^3+^ enrichment is associated with the substitution of Ni^2+^ by Mg^2+^, which is consistent with previous reports on cation-alloyed metal oxides.^29^ The slightly increased Ni^3+^ ratio is accompanied by an increase in Ni vacancies, which undoubtedly contributes to the improvement of hole concentration.^30^

**Table 1:** Summary of Ni Ion Changes of Mg_*y*_Ni_1–*y*_O_*x*_ Films from XPS Spectra

Samples	Ni^2+^Atomic (%)	Ni^3+^Atomic (%)	Ni^3+^/Ni^2+^
NiO_*x*_	27.98	72.02	2.57
Mg_0.02_Ni_0.98_O_*x*_	24.31	75.69	3.11
Mg_0.03_Ni_0.97_O_*x*_	21.41	78.59	3.67
Mg_0.04_Ni_0.96_O_*x*_	20.20	79.80	3.95

The position of the Fermi level of the HIL significantly affects the hole injection barrier. The work function can be evaluated from Kelvin probe force microscopy (KPFM) images. As shown in Figure 2a–d, the contact potential difference between sample and tip (*V*_CPD_) progressively increases with Mg composition. This trend is visually summarized in Figure 2e, and the *V*_CPD_ saturates in the Mg_0.03_Ni_0.97_O_*x*_ and Mg_0.04_Ni_0.96_O_*x*_ samples. The *V*_CPD_ of pure NiO_*x*_ (∼550 mV) corresponds to a low work function, which can be calculated as follows:

$${\mathrm{eV}}_{\mathrm{CPD}}=\varphi_{\mathrm{sample}}-\varphi_{\mathrm{tip}}$$

where φ_sample_ and φ_tip_ are the work functions of the sample and tip, respectively. According to the φ_tip_ of 4.65 eV (Figure S3), the work functions are calculated as 5.20 eV (NiO_*x*_), 5.40 eV (Mg_0.02_Ni_0.98_O_*x*_), 5.49 eV (Mg_0.03_Ni_0.97_O_*x*_), and 5.54 eV (Mg_0.04_Ni_0.96_O_*x*_) (Figure 2f). Although Mg_0.04_Ni_0.96_O_*x*_ exhibits the deepest work function, Mg_0.03_Ni_0.97_O_*x*_ (5.49 eV) more closely matches the TFB HOMO level (5.50 eV), facilitating hole injection.^31^ The deeper work function of Mg:NiO_*x*_ reduces the hole injection barrier into TFB, attributable to MgO’s wider bandgap (∼7.8 eV^32^) and its VBM lying 0.9 eV deeper than that of NiO_*x*_.^33^ This observation aligns with the trend that Ni^3+^/Ni^2+^ increases with Mg composition, arising from the elevated work function associated with high-valent cations exhibiting enhanced electronegativity and electronic chemical potential.^34^ Meanwhile, the increased work function enhances hole occupancy probability, which is further supported by ultraviolet photoelectron spectroscopy (UPS) and ultraviolet–visible absorption (UV–vis) spectra (Figure S4). The valence band region reveals a decrease in the energy difference Δ*E*_VB_ between the Fermi level and the VBM from 0.64 eV (NiO_*x*_) to 0.55 eV (Mg_0.03_Ni_0.97_O_*x*_). According to the Boltzmann statistical distribution law of carriers in semiconductors, the hole concentration in valence band can be expressed as^35^

$$p_{0}=N_{v}\exp\left(-\frac{\Delta E_{\mathrm{VB}}}{k_{0}T}\right)$$

where *N*_v_ is the effective state density of the valence band, *k*_0_ is the Boltzmann constant, and *T* is the temperature. The decrease in Δ*E*_VB_ suggests an increased hole concentration in the valence band of Mg_0.03_Ni_0.97_O_*x*_ film, which is consistent with the literature.^36^ The deepened work function of Mg_0.03_Ni_0.97_O_*x*_ facilitates hole injection and transport due to the reduced hole injection barrier and enhanced hole occupancy probability. This provides a basis for more efficient radiative recombination in the QLEDs.

**Figure 2:**
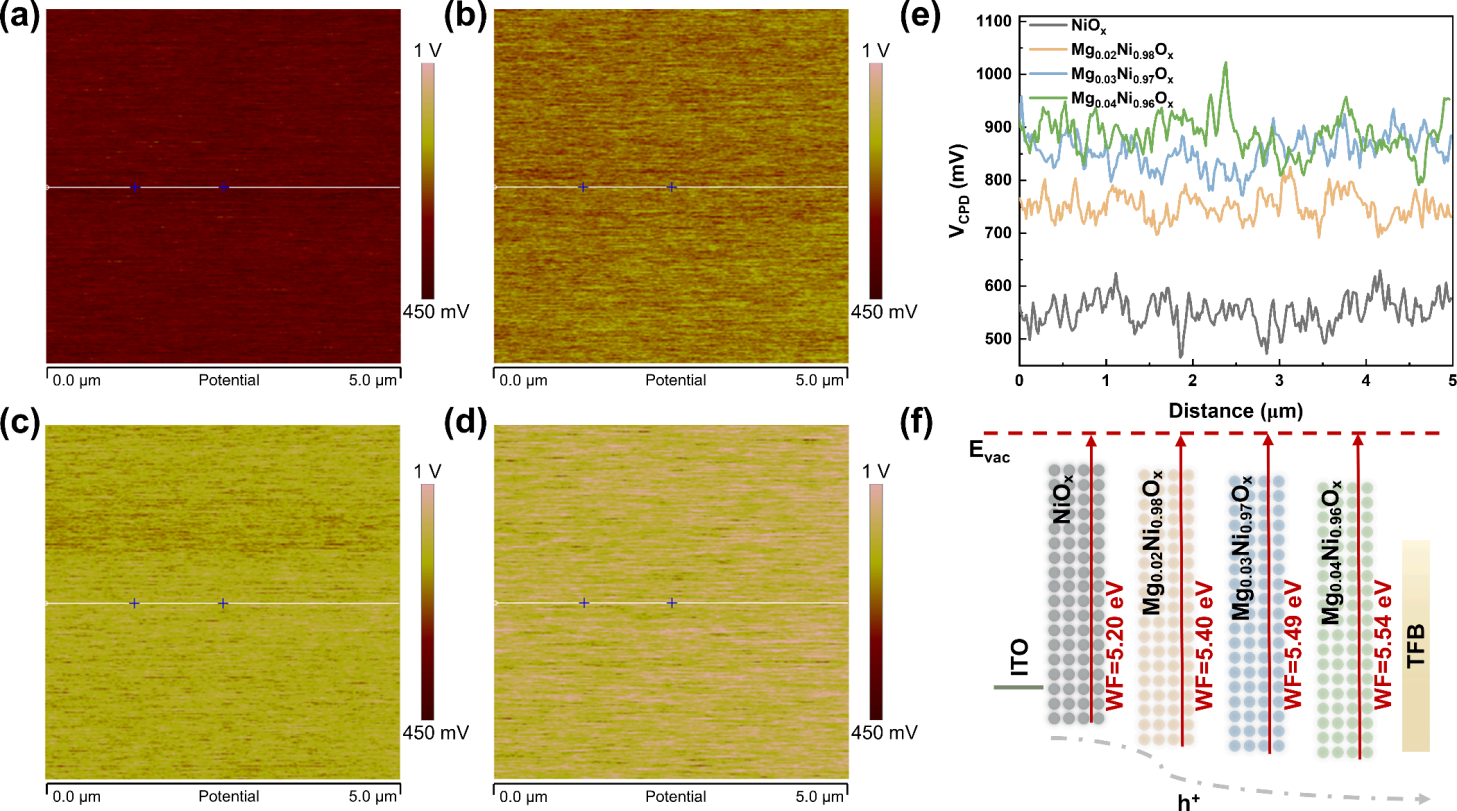
Effect of Mg alloying on the band structure. KPFM images of (a) NiO_*x*_, (b) Mg_0.02_Ni_0.98_O_*x*_, (c) Mg_0.03_Ni_0.97_O_*x*_, and (d) Mg_0.04_Ni_0.96_O_*x*_ films. (e) Contact potential difference (*V*_CPD_) profiles. (f) Schematic diagram of work function deepening of Mg_*y*_Ni_1–*y*_O_*x*_ films.

Studies have demonstrated that imperfect interfacial contact induces nonradiative recombination centers and impedes carrier transport.^37^ In the Mg:NiO_*x*_ films, Mg incorporation preserves the surface morphology (Figure S5) and reduces the roughness (Figure S6), offering an optimal substrate for intimate HIL/HTL interfacial contact and enabling uniform deposition of subsequent HTL and QDs layer. The dynamic process of charge transfer is investigated through time-resolved photoluminescence (TrPL) and steady-state PL spectra (Figure S7), and the results reveal that the exciton lifetime of the Mg_0.03_Ni_0.97_O_*x*_ sample is significantly prolonged to 19.48 ns (13.88 ns in the NiO_*x*_ sample), accompanied by a concomitant enhancement in PL emission. The simultaneous extension of exciton lifetime and enhancement of PL intensity arise from optimized HIL/HTL interface engineering, consistent with established mechanisms of suppressed exciton quenching.^38^

While Mg composition enhances p-type characteristics and marginally increases hole concentration, the hole current enhancement remains severely limited, as demonstrated by conductive atomic force microscopy (C-AFM) of films and current density–voltage (*J*–*V*) analyses of the corresponding devices (Figure S8). This limitation likely originates from Mg^2+^-induced lattice distortions and the accompanying enhancement of carrier scattering.^39^ The low hole current directly indicates compromised transport characteristics, which restricts hole injection into QDs. To achieve the expected improvement of hole current in high-performance QLEDs, Mg_0.03_Ni_0.97_O_*x*_ films were treated with O_3_ in an atomic layer deposition (ALD) system, which enables precise control over O_3_ composition and exposure durations through deposition cycle modulation. After treatment with the O_3_ for varying durations, the Mg_0.03_Ni_0.97_O_*x*_ films exhibit uniform current distributions and an increase in current magnitude with treatment duration in the C-AFM images (Figure 3a–d). Notably, the inset reveals that the current of the pristine Mg_0.03_Ni_0.97_O_*x*_ film is 40 nA, while the 2 min O_3_ treatment significantly enhances it to 150 nA. This increase indicates improved hole transport through the HIL, attributed to enhanced conductivity. The enhancement of hole transport performance is further confirmed by *J*–*V* characteristics of a single-layer device (Figure 3e). As the duration of the O_3_ treatment increases, the current through the Mg_0.03_Ni_0.97_O_*x*_ film is significantly enhanced. The conductivity of the Mg_0.03_Ni_0.97_O_*x*_ film can be calculated as follows:^40^

$$\sigma =\frac{d}{\mathrm{AR}}$$

where *A* is the device area (0.06 cm^2^), *d* is the HIL thickness (∼100 nm), and *R* is the resistance. The conductivities of Mg_0.03_Ni_0.97_O_*x*_ films treated with O_3_ for 0, 1, 2, and 3 min are 8.65 × 10^–7^, 1.01 × 10^–6^, 1.33 × 10^–6^, and 1.54 × 10^–6^ S cm^–1^, respectively. The conductivity of the Mg_0.03_Ni_0.97_O_*x*_-2 min O_3_ film increases by an order of magnitude compared with the pristine Mg_0.03_Ni_0.97_O_*x*_ film. Enhanced conductivity in the HIL minimizes losses during hole transport, facilitating efficient hole delivery to the HIL/HTL interface, thereby improving the hole injection efficiency.

**Figure 3:**
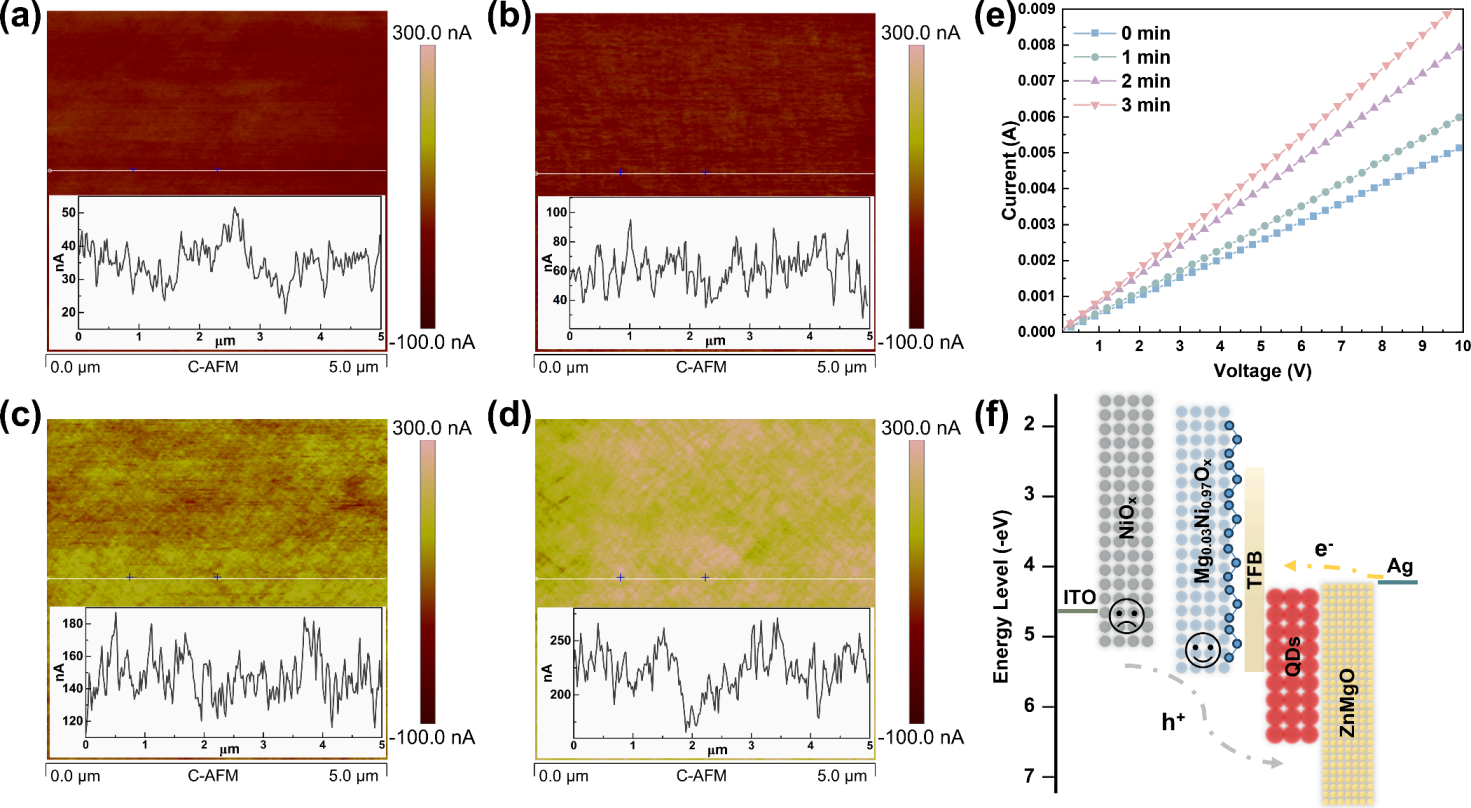
O_3_ treatment on Mg_0.03_Ni_0.97_O_*x*_ films. C-AFM images of Mg_0.03_Ni_0.97_O_*x*_ films with (a) 0, (b) 1, (c) 2, and (d) 3 min of O_3_ treatment. (e) Current–voltage characteristics of Mg_0.03_Ni_0.97_O_*x*_ films with different O_3_ exposure durations (ITO/Mg_0.03_Ni_0.97_O_*x*_/Ag). (f) Energy band diagram of QLEDs based on NiO_*x*_ or Mg_0.03_Ni_0.97_O_*x*_ HILs.

Furthermore, the space charge limited current (SCLC) model demonstrates that the Mg_0.03_Ni_0.97_O_*x*_-2 min O_3_ device exhibits a lower trap-filled limit voltage (*V*_TFL_ = 1.17 V) compared to pristine Mg_0.03_Ni_0.97_O_*x*_ device (*V*_TFL_ = 2.80 V), indicating the reduction in trap density (Figure S9).^41^ The suppressed trap density mitigates carrier annihilation caused by nonradiative recombination at the HIL/HTL interface, thereby enhancing hole extraction efficiency. The low intrinsic conductivity of NiO_*x*_ and its shallow work function accompanied by a significant hole injection barrier at the NiO_*x*_/TFB interface (Figure 3f) critically limit hole injection efficiency, constituting the primary factor for the poor performance of QLEDs. The incorporation of Mg deepens the work function to 5.49 eV and reduces the energy offset between the HIL and HTL. Subsequent O_3_ treatment compensates for the insufficient hole current by enhancing the conductivity, thereby promoting enhanced exciton recombination within the QD layer.

The solution-processed QLEDs were fabricated with the following structure: ITO/Mg_0.03_Ni_0.97_O_*x*_-2 min O_3_/TFB/CdSe QDs/ZnMgO/Ag (Figure 4a). The corresponding cross-sectional transmission electron microscopy (TEM) image demonstrates excellent film quality and intimate interfacial contact (Figure 4b). The thicknesses of the Mg_0.03_Ni_0.97_O_*x*_, TFB, QDs, and ZnMgO layers are 100, 10, 15, and 75 nm, respectively. The detailed QD information is provided in Figure S10. The current density of the Mg_0.03_Ni_0.97_O_*x*_-2 min O_3_ device is significantly enhanced, consistent with the improved hole conduction in the Mg_0.03_Ni_0.97_O_*x*_ film due to the O_3_ treatment (Figure 4c). Coupled with a reduced turn-on voltage (*V*_T_) of 2.07 V, this demonstrates an enhanced hole injection efficiency. The champion device, exhibiting an EL peak at 620 nm with CIE chromaticity coordinates of (0.68, 0.33) (Figure S11), achieves a peak current efficiency (CE) of 17.85 cd A^–1^ and a peak power efficiency (PE) of 11.23 lm W^–1^ (Figure 4e), corresponding to 54% and 171% enhancements over the pristine NiO_*x*_-based QLEDs (11.56 cd A^–1^, 4.15 lm W^–1^), respectively. This substantial PE enhancement stems from both increased CE and a reduced operating voltage at equivalent CE, consistent with the relationship between PE ∝ CE/*V*, leading to significantly lower power consumption. The lower current density and higher *V*_T_ (4.69 V) of the pristine NiO_*x*_-based QLEDs are primarily due to insufficient hole current and the significant hole injection barrier. Optimizing Mg composition to 0.03 effectively lowers the hole injection barrier, consequently reducing the *V*_T_ and aligning with prior findings that decreased injection barriers lead to earlier turn-on.^42^ The higher *V*_T_ (3.71 V) of unalloyed NiO_*x*_-2 min O_3_ QLEDs compared to Mg_0.03_Ni_0.97_O_*x*_-2 min QLEDs (2.07 V) further confirms the essential role of Mg alloying in increasing the work function (Figure S12). Subsequent O_3_ treatment notably increases the hole current and curtails energy loss via enhanced conductivity. The impacts of Mg alloying and O_3_ treatment on hole injection efficiency are substantiated through the systematic variation of Mg compositions and O_3_ exposure durations (Figure S13). The increases in PE and CE directly reflect the enhanced carrier utilization and increased radiative exciton recombination in Mg_0.03_Ni_0.97_O_*x*_-2 min O_3_ QLEDs (Table S2). Crucially, despite higher hole current in the Mg_0.03_Ni_0.97_O_*x*_-3 min of the O_3_ device (Figure 3e), diminished film transmittance under extended O_3_ exposure impaired photon outcoupling (Figure S14), leading to reduced luminance and lower efficiency (Figure S13). The combined reduction of injection barrier and augmentation of hole current collectively elevate hole injection efficiency, rationalizing the exceptional performance of QLEDs. Importantly, the efficiency roll-off behavior is mitigated through synergistic optimization of hole transport dynamics and suppression of interfacial nonradiative losses at the HIL/HTL.

**Figure 4:**
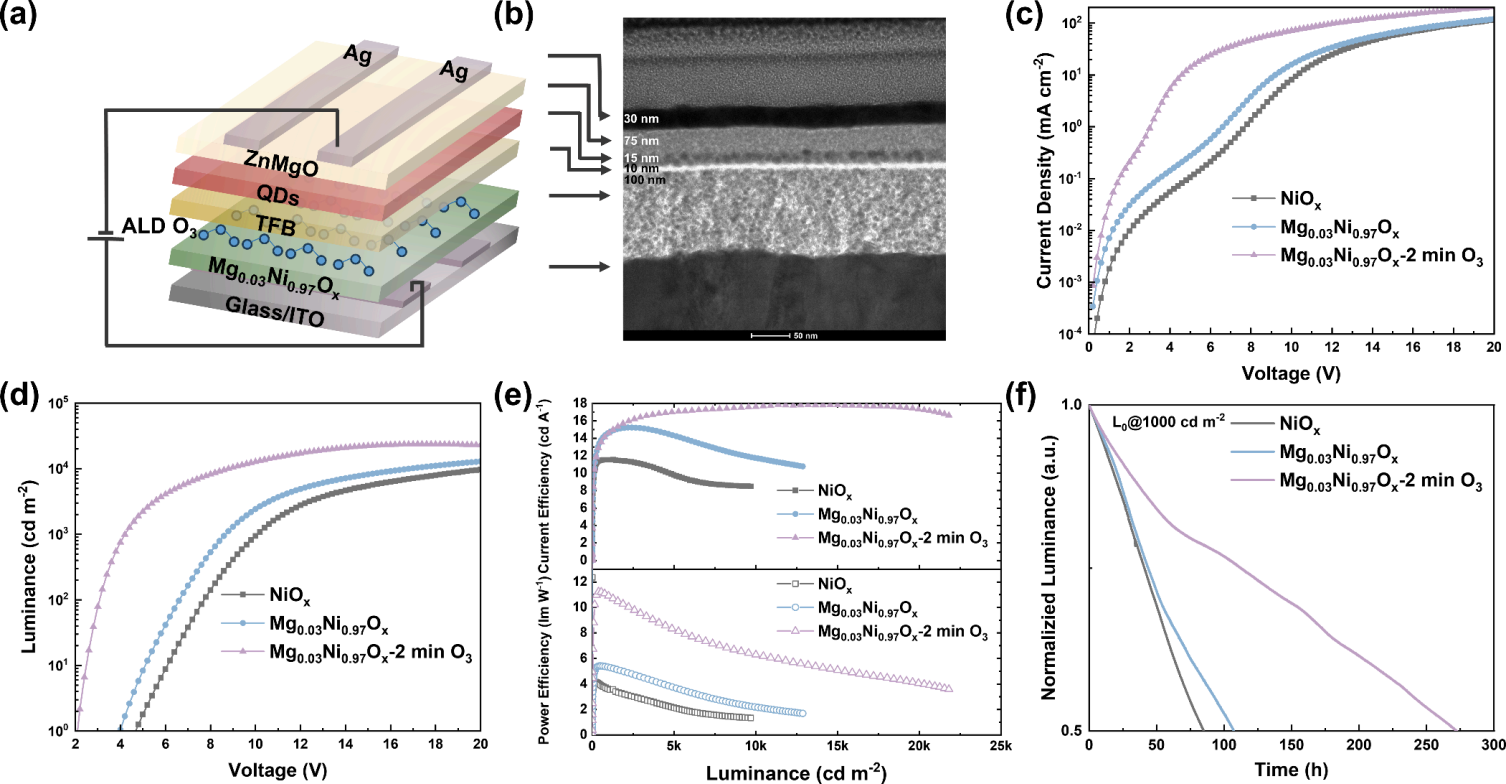
Performance of QLEDs with different HILs. (a) The device structure and (b) cross-sectional TEM image of the QLEDs. (c) *J*–*V* characteristics, (d) luminance–voltage (*L*–*V*) characteristics, and (e) current efficiency–luminance (CE–*L*), and power efficiency (PE–*L*) curves of QLEDs with different HILs. (f) Luminance versus operation time of the device driven at an initial luminance of 2000 cd m^–2^ of the device. The lifetime of device is simulated at an initial luminance of 1000 cd m^–2^ based on the relation (*L*_0_)^*n*^ × *T*_50_ = *C* (*n* = 1.8).

Furthermore, the operational stability of the Mg_0.03_Ni_0.97_O_*x*_-2 min O_3_ QLEDs demonstrates an exceptional performance. Based on the empirical scaling law, (*L*_0_)^*n*^ × *T*_50_ *= C* with the acceleration factor *n* = 1.8,^43^ the device exhibited a 272 h *T*_50_ lifetime (*L*_0_ = 1000 cd m^–2^), which is over 1.5-fold and 2.2-fold that of the Mg_0.03_Ni_0.97_O_*x*_-based QLEDs (107 h) and the NiO_*x*_-based QLEDs (84 h), respectively (Figure 4f). It is worth mentioning that the *T*_50_ of the champion QLEDs shows a ∼13-fold increase compared to that of the PEDOT:PSS-based QLEDs (19 h) (Figure S15). The prolonged lifetime and enhanced performance are attributed to the synergistic effects of Mg alloying and the O_3_ treatment, which collectively optimize hole injection efficiency through a reduced injection barrier and improved hole conductivity.

In summary, a synergistic approach integrating Mg alloying and ALD O_3_ treatment to NiO_*x*_ NPs was proposed to fabricate efficient and stable NiO_*x*_-based QLEDs. The effect of Mg composition on the modulation of the band alignment and the effect of O_3_ treatment on the current enhancement led to an improvement in the hole injection efficiency. Specifically, the work function of the Mg_0.03_Ni_0.97_O_*x*_ film was deepened to 5.49 eV and the hole conductivity was significantly improved by an order of magnitude. This dual modulation synergistically enhanced hole injection yielded Mg_0.03_Ni_0.97_O_*x*_-2 min O_3_ QLEDs with peak CE of 17.85 cd A^–1^ and PE of 11.23 lm W^–1^. Notably, the improved hole utilization extended operational stability and demonstrated a *T*_50_ lifetime of 272 h at 1000 cd m^–2^, representing a 2.2-fold (84 h) improvement compared to NiO_*x*_-based QLEDs. Our findings establish Mg–O_3_ co-optimization as a critical strategy for advancing NiO_*x*_-based QLEDs’ performance.

## Supplementary Material



## Notes

Thermal decomposition behavior and wide survey XPS spectra; KPFM images and UPS spectra; SEM and AFM images; TrPL and PL spectra; C-AFM images of Mg_*y*_Ni_1–*y*_O_*x*_ films and *J*–*V* characteristics of hole-only device; absorption and PL spectra of QDs; EL spectrum and CIE coordinates; optical transmission spectra of Mg_0.03_Ni_0.97_O_*x*_ films; *J*–*V*–*L* curves and CE-L and PE-L curves of NiO_*x*_-based QLEDs and PEDOT:PSS-based QLEDs; fitting of TrPL (PDF)
