# Supplementary material for: Mg-Incorporated Nickel Oxide Hole Injection Layer for Stable and Efficient Quantum Dot Light-Emitting Diodes
Source: J Phys Chem Lett. 2025 Aug 28;16(36):9242–8. doi: 10.1021/acs.jpclett.5c02298 (PMC12434719; doi:10.1021/acs.jpclett.5c02298)
Supplement: Supplementary file 1 [file jz5c02298_si_001.pdf]

Supporting Information

Mg Incorporated Nickel Oxide Hole Injection Layer for Stable and Efficient Quantum Dot Light-Emitting Diodes

Meng-Wei Wang, Ting Ding, Yin-Man Song, Hang Liu, Jing Jiang, Pei-Li Gao*, Kar Wei Ng*, Shuang-Peng Wang*

Institute of Applied Physics and Materials Engineering, University of Macau, Taipa, Macao SAR 999078, China

* Corresponding author:

Pei-Li Gao (peiligao@um.edu.mo),

Kar Wei Ng (billyng@um.edu.mo),

Shuang-Peng Wang (spwang@um.edu.mo).

Experimental Methods

Materials. Nickel acetate tetrahydrate ($Ni(CH_3COO)_2 \cdot 4H_2O$) and magnesium acetat e tetrahydrate ($Mg(CH_3COO)_2 \cdot 4H_2O$) were purchased from Sigma-Aldrich and Alad din Reagent, respectively. Poly((9,9-dioctylfluorenyl-2,7-diyl)-co-(4,4′-(N-(4-secbutylp henyl)diphenylamine)) (TFB) was purchased from Xi'an Yuri Solar Co., Ltd. Red QDs were purchased from Poly OptoElectronics Co., Ltd. $Ni(CH_3COO)_2 \cdot 4H_2O$ and ethanolamine were dissolved in ethanol (0.6 mol L^{-1}) at a molar ratio of 1:1. $Mg(CH_3COO)_2 \cdot 4H_2O$ and $Ni(CH_3COO)_2 \cdot 4H_2O$ were mixed in the selected ratios. A dark green transparent solution was obtained by stirring at room temperature in air for 12 h. The precursor was aged for 24 h and then used to prepare $Mg_yNi_{1-y}O_x$ films. To prepare ZnMgO NPs, 0.02375 mol $Zn(OAc)_2 \cdot 2H_2O$ (99%, Alfa Aesar, China) and 0.00125 mol $Mg(OAc)_2 \cdot 4H_2O$ (99%, Alfa Aesar, China) were dissolve d in 150 mL ethanol contained in a condenser-fitted three-necked flask. The soluti on underwent stirring at 80 ^{o}C for 70 min for full dispersion. Separately, 2 g KO H powder (99%, Sinopharm Chemical, China) was rapidly added to 20 mL ethano l and sonicated for 10 min. This KOH solution was dripped into the metal precur sor solution. After reacting for 5 min, the precipitated material was purified by w ashing with ethanol and n-hexane, followed by evacuation for 1 h. The synthesize d $Zn_{0.95}Mg_{0.05}O$ was dispersed in ethanol (30 mg mL^{-1}) and combined with ethano lamine for later use.

Device Fabrication. To prepare QLEDs, ITO-coated glass substrates (45 ohm sq^{-1}) were first ultrasonically rinsed with detergent water, deionized water, and ethanol for 30 min, respectively. After that, the substrates were exposed to UV-ozone treatment for 10 min to make ITO hydrophilic. The $Mg_yNi_{1-y}O_x$ precursor solution was filtered using a 0.45 μm organic filter and spin-coated at 3000 rpm for 40 s. It was first soft annealed in air at 110 ^{o}C for 15 min, then transferred to a muffle furnace and heated to 400 ^{o}C for 1 h. After the

crystallization process, the substrate was transferred to a glove box filled with nitrogen. Subsequently, the $Mg_yNi_{1-y}O_x$ film was subjected to ALD O_3 treatment for different cycles. The ALD O_3 treatment parameters: substrate temperature at room temperature; chamber pressure at 2.6 x 10^{-1} Torr; purge gas at 99.999% nitrogen; and 1, 2, and 3 min O_3 treatments consisting of 4, 8, and 12 cycles, respectively. TFB solution (dissolved in chlorobenzene at a concentration of 8 mg mL^{-1}) was spin-coated at 3000 rpm for 40 s and annealed at 150 ^oC for 15 min. QDs solution (dissolved in octane at a concentration of 20 mg mL^{-1}) was then spin-coated at 3000 rpm for 40 s and annealed at 90 ^oC for 10 min. ZnMgO solution was spin-coated at 3000 rpm for 40 s and annealed at 60 ^oC for 10 min. Finally, the Ag electrode was obtained by thermal evaporation.

Characterization. The crystal structure and diffraction pattern of $Mg_yNi_{1-y}O_x$ NPs were obtained by X-ray diffraction (XRD; Smartlab, Rigaku, Japan). The composit ion of the surface chemical state and the band structure were obtained by X-ray p hotoelectron spectroscopy and Ultraviolet photoelectron spectroscopy, respectively (XPS, UPS; ESCALAB Xi^+, Thermo Fisher Scientific, USA). The surface morphol ogy of $Mg_yNi_{1-y}O_x$ films were obtained by scanning electron microscopy (SEM; Si gma, Zeiss, Germany). The roughness was characterized by atomic force microsco py (AFM, Dimension Icon, Bruker, Germany). Photoluminescence (PL) and time-re solved PL (TrPL) spectra were measured using a fluorescence spectrometer (FS5-T CSPC, Edinburgh Instruments Ltd.). Ultraviolet-visible absorption (UV-Vis) spectra and optical transmission spectra were measured using a UV/VIS/NIR spectrophoto meter (V770 Jasco). The device performances, including current density, luminance, current efficiency, and power efficiency, were measured by a constant current so urce (Model 2400, Keithley, USA) combined with a luminance meter (LS160, Ko nica Minolta, Japan).

Figures and Captions

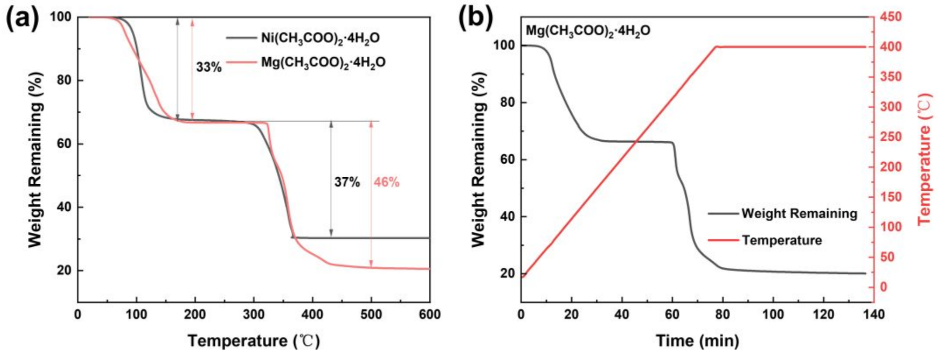

Figure S1. Thermal decomposition behavior. (a) TGA spectra of $Ni(CH_3COO)_2{\cdot}4H_2O$ and $Mg(CH_3COO)_2{\cdot}4H_2O$ powders and (b) Treat $Mg(CH_3COO)_2{\cdot}4H_2O$ powder in muffle furnace at 400 ^oC for 1 hour to ensure that the experimental conditions are sufficient to complete the complete pyrolysis of $Mg(CH_3COO)_2{\cdot}4H_2O$.

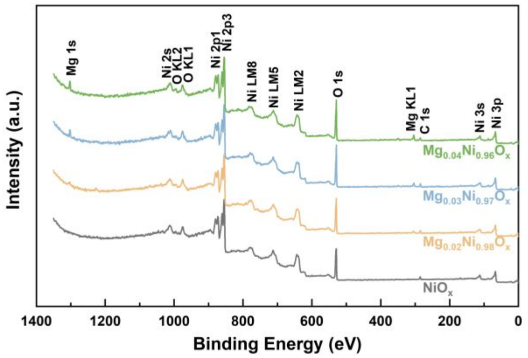

Figure S2. Wide survey XPS spectra of $Mg_yNi_{1-y}O_x$ NPs.

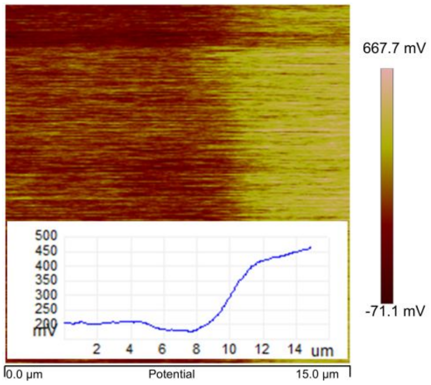

Figure S3. KPFM images and contact potential difference (V_{CPD}) profiles of AlSiAu standard sample.

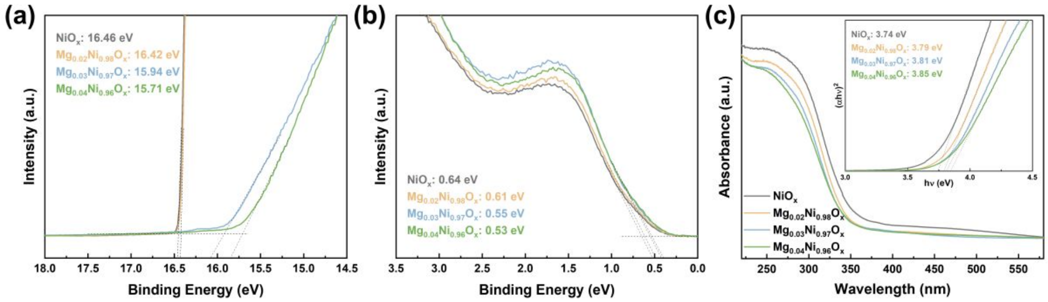

Figure S4. UPS spectra of $Mg_yNi_{1-y}O_x$ films in the (a) cut-off and (b) valence band regions. (c) UV-Vis absorption spectra and inset is Tauc's plot for band gap determination of HILs. The optical band gap energy (E_g) of the $Mg_yNi_{1-y}O_x$ thin films was determined by $(\alpha h\nu)^2 = A(h\nu\text{-}E_g)^n$, where α is absorption coefficient, hν is the photon energy, E_g is the optical band gap energy, and A is a constant. A value of n = 1/2 is found to be most suitable for direct band gap NiO_x thin film.

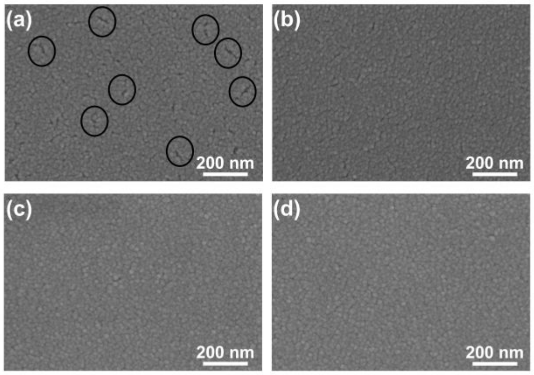

Figure S5. SEM images of (a) NiO_x, (b) $Mg_{0.02}Ni_{0.98}O_x$, (c) $Mg_{0.03}Ni_{0.97}O_x$, and (d) $Mg_{0.04}Ni_{0.96}O_x$ films.

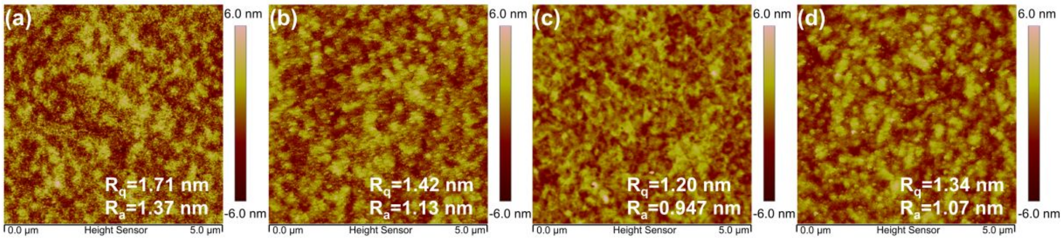

Figure S6. AFM images of (a) NiO_x, (b) $Mg_{0.02}Ni_{0.98}O_x$, (c) $Mg_{0.03}Ni_{0.97}O_x$, and (d) $Mg_{0.04}Ni_{0.96}O_x$ films.

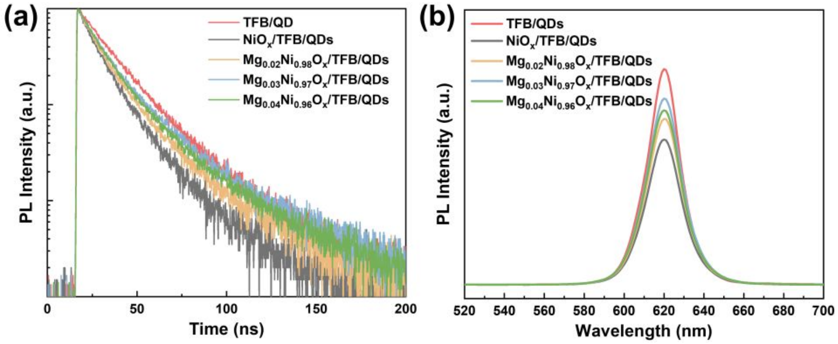

Figure S7. (a) TrPL curves and (b) PL spectra of TFB/QDs, NiO_x or $Mg_{0.02}Ni_{0.98}O_x$ or $Mg_{0.03}Ni_{0.97}O_x$ or $Mg_{0.04}Ni_{0.96}O_x$/TFB/QDs.

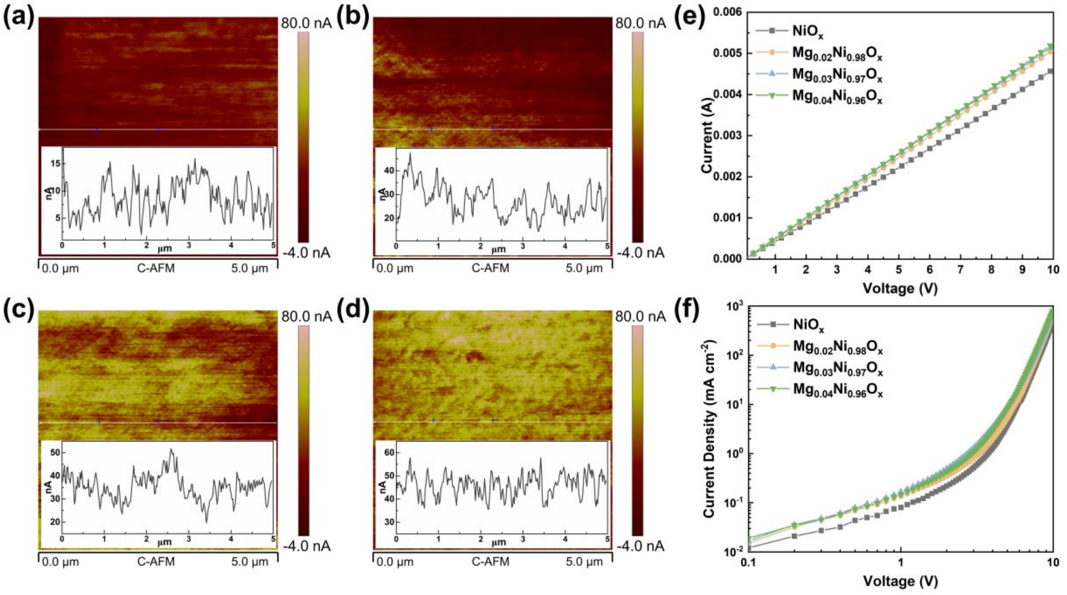

Figure S8. Effect of Mg alloying on the current. C-AFM images of (a) NiO_x, (b) $Mg_{0.02}Ni_{0.98}O_x$, (c) $Mg_{0.03}Ni_{0.97}O_x$, and (d) $Mg_{0.04}Ni_{0.96}O_x$ films. (e) current-voltage characteristics of single-layer devices (ITO/$Mg_yNi_{1-y}O_x$ with different Mg compositions/Ag). (f) J-V characteristics of hole-only devices (ITO/$Mg_yNi_{1-y}O_x$ with different Mg compositions/TFB/QDs/TCTA/MoO_3/Ag).

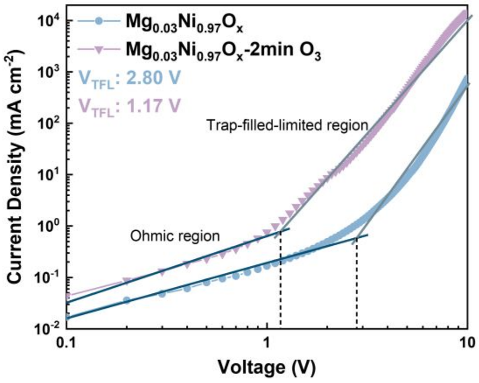

Figure S9. J-V characteristics of hole-only device (ITO/HILs/TFB/QDs/TCTA/MoO_3/Ag).

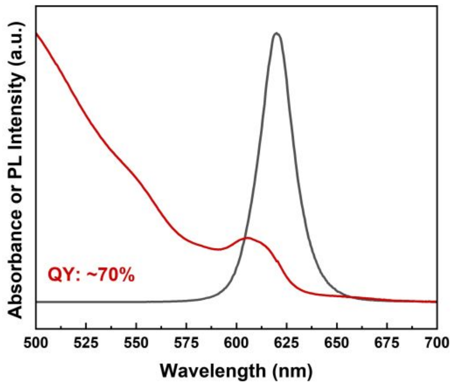

Figure S10. Absorption spectra (red line) and PL spectra (black line) of QDs. The QDs employed in this work are based on CdSe/ZnS core-shell structure. The emission peak is centered at 620 nm with corresponding photoluminescence quantum yield (PLQY) of ~70%.

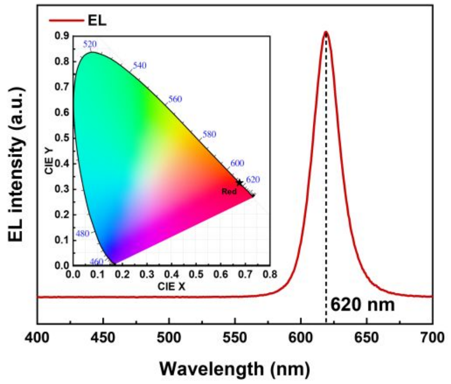

Figure S11. The EL spectrum of the device. The CIE coordinates are X=0.68, Y=0.33.

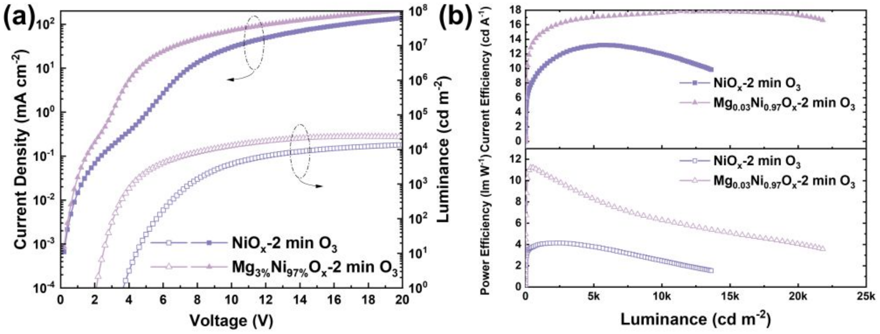

Figure S12. Device performance. (a) J-V-L curves and (b) CE-L and PE-L curves of NiO_x-2 min O_3 QLEDs and $Mg_{0.03}Ni_{0.97}O_x$-2 min O_3 QLEDs.

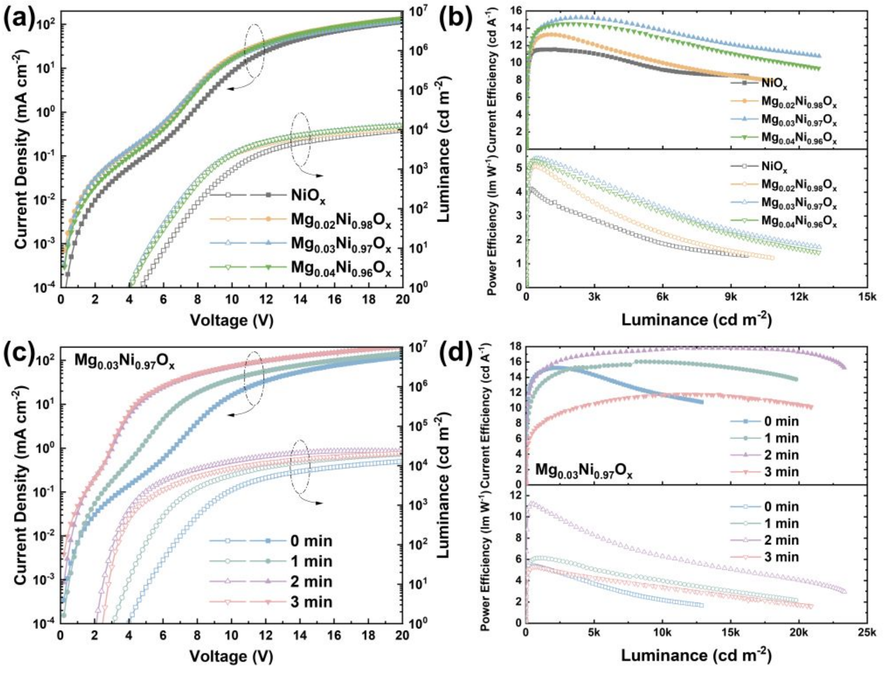

Figure S13. Device performance. (a) J-V-L curves and (b) CE-L and PE-L curves of NiO_x devices with different Mg compositions. (c) J-V-L curves and (d) CE-L and PE-L curves of $Mg_{0.03}Ni_{0.97}O_x$ devices with different O_3 exposure durations.

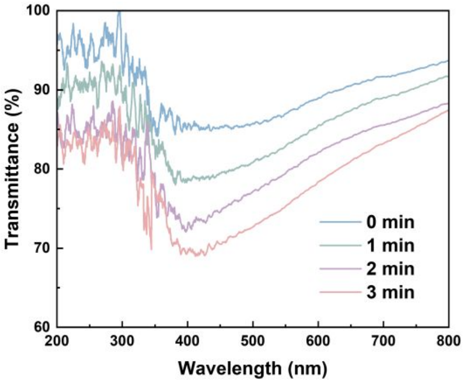

Figure S14. Optical transmission spectra of $Mg_{0.03}Ni_{0.97}O_x$ films at different O_3 exposure durations.

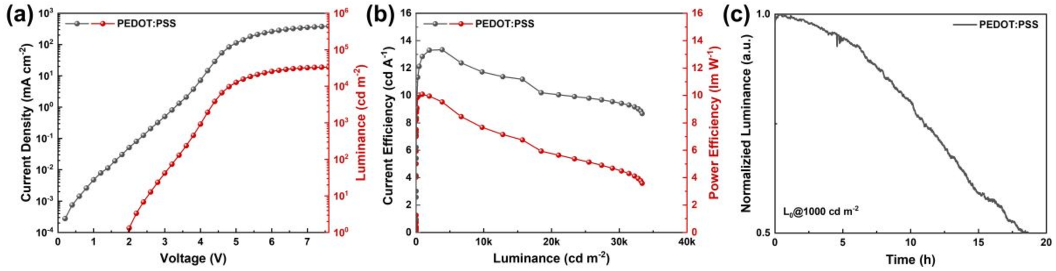

Figure S15. Performance of PEDOT:PSS-based QLEDs. (a) J-V-L curves, (b) CE-PE-L curves, and (c) luminance versus operation time of the device driven at an initial luminance of 2000 cd m^{-2} of the device. The lifetime of device is simulated at an initial luminance of 1000 cd m^{-2} based on the relation $(L_0)^n \times T_{50} = C$ (n = 1.8).

Table S1. Fitted TrPL parameters of TFB/QDs on different HILs: NiO_x, $Mg_{0.02}Ni_{0.98}O_x$, $Mg_{0.03}Ni_{0.97}O_x$, and $Mg_{0.04}Ni_{0.96}O_x$.

Samples	A_1	τ_1 (ns)	A_2	τ_2 (ns)	τ (ns)
TFB/QDs	0.77	15.28	0.23	30.22	20.89
NiO_x/TFB/QDs	0.61	7.66	0.51	17.15	13.88
$Mg_{0.02}Ni_{0.98}O_x$/TFB/QDs	0.71	9.34	0.36	22.60	16.71
$Mg_{0.03}Ni_{0.97}O_x$/TFB/QDs	0.72	10.61	0.34	26.83	19.48
$Mg_{0.04}Ni_{0.96}O_x$/TFB/QDs	0.71	10.68	0.29	26.73	18.72

The TrPL curves fit a double exponential model. The average exciton lifetime (τ) is obtained as follows:

$$y=A_1 e^{-t/\tau_1}+A_2 e^{-t/\tau_2}+y_0$$

$$\tau=\frac{\sum A_i \tau_i^2}{\sum A_i \tau_i}$$

where A_1 and A_2 are the amplitude coefficients of lifetimes.

Table S2. EL performance of QLEDs with different HILs.

HIL	V_T (V)	Peak CE (cd A^{-1})	Peak L (cd m^{-2})	Peak PE (lm W^{-1})	T_{50}@1000 cd m^{-2} (hours)	Peak EQE (%)
NiO_x	4.69	11.56	9688	4.15	84	6.97
$Mg_{0.03}Ni_{0.97}O_x$	4.00	15.24	12871	5.42	107	9.19
$Mg_{0.03}Ni_{0.97}O_x$-2 min O_3	2.07	17.85	23679	11.23	272	10.76

EQE is calculated as follows:[1]

$$EQE=\frac{1.18 * \text{current efficiency} * \pi * \text{Emission wavelength}}{\text{Visual constant} * 10000}$$

(1) Forrest, S. R.; Bradley, D. D.; Thompson, M. E. Measuring the Efficiency of Organic Light-Emitting Devices. *Adv. Mater.* **2003**, *15*, 1043-1048.
